# Risk of breast cancer according to clinicopathologic features among long-term survivors of Hodgkin's lymphoma treated with radiotherapy

**DOI:** 10.1038/sj.bjc.6605877

**Published:** 2010-09-14

**Authors:** G M Dores, W F Anderson, L E Beane Freeman, J F Fraumeni, R E Curtis

**Affiliations:** 1 Medical Service, Department of Veterans Affairs Medical Center, 921 N.E. 13th Street, Oklahoma City, OK 73104, USA; 2 Division of Cancer Epidemiology and Genetics, National Cancer Institute, National Institutes of Health, Department of Health and Human Services, 6120 Executive Boulevard, Bethesda, MD 20892, USA

**Keywords:** Hodgkin's lymphoma, second breast cancer, radiation-related breast cancer

## Abstract

**Background:**

It is unknown whether breast cancer (BC) characteristics among young women treated with radiotherapy (RT) for Hodgkin's lymphoma (HL) differ from sporadic BC.

**Methods:**

Using population-based data, we calculated BC risk following HL according to clinicopathologic features.

**Results:**

Compared with BC in the general population, risks of oestrogen receptor (ER)-positive/progesterone receptor (PR)-positive and ER-negative/PR-negative BC in young, irradiated HL survivors were increased five-fold (95% confidence interval (CI)=3.81–6.35) and nine-fold (95% CI=6.93–12.25), respectively. Among 15-year survivors, relative risk of ER-negative/PR-negative BC exceeded by two-fold (*P*=0.002) than that of ER-positive/PR-positive BC.

**Conclusion:**

Radiotherapy may disproportionately contribute to the development of BC with adverse prognostic features among young HL survivors.

Young women treated with radiotherapy (RT) for Hodgkin's lymphoma (HL) have an elevated risk of developing breast cancer (BC) compared with the general population (Dores *et al*, 2002; Hodgson *et al*, 2007). Hospital-based, case–control studies have addressed whether features of radiation-related BC differ from primary BC (Yahalom *et al*, 1992; Gaffney *et al*, 2001; Janov *et al*, 2001; Castiglioni *et al*, 2007; Sanna *et al*, 2007). As the largest report included fewer than 60 HL cases (Castiglioni *et al*, 2007), and only one focused on females diagnosed with HL at age 40 years or younger (Janov *et al*, 2001), statistical power to evaluate risk of BC characteristics compared with sporadic BC may have been limited in these studies. To date, no population-based series of young HL survivors has assessed BC risk according to clinicopathologic characteristics. Therefore, we sought to assess risk of BC subtypes among long-term survivors of HL treated with RT compared with BC in the general population.

## Materials and Methods

We evaluated the risk of invasive BC among female 5-year survivors of HL diagnosed before 35 years of age who received RT as part of initial therapy for HL and who were reported to one of nine cancer registry areas of the Surveillance, Epidemiology and End Results (SEER) Programme in the United States during 1973–2000 and followed through 2005 (SEER-9, 2008). The SEER Programme classifies information on histology and topography according to the third edition of the International Classification of Diseases for Oncology (ICD-O-3) (Fritz *et al*, 2000). Most clinicopathologic variables were defined as in previous studies (Anderson *et al*, 2001, 2002) and are detailed in Table 1. The SEER Programme began collecting information on tumour size and regional lymph node (LN) involvement in 1988, and oestrogen receptor (ER) and progesterone receptor (PR) status in 1990. For consistency, analyses including data on ER and PR status, tumour size, grade and LN involvement were limited to BC cases diagnosed in 1990 or later.

Standardised incidence ratios (SIRs) were calculated as the ratio of observed (Obs)-to-expected number of second or higher order invasive BC using methods previously described (Curtis and Ries, 2006). Age-, race- and calendar year-specific incidence rates (IRs) of invasive BC were computed for each group of BC clinicopathologic characteristics from the general SEER population, as specified in Table 1. We calculated exact, two-sided, Poisson-based 95% confidence intervals (CIs). Multivariate analyses using Poisson regression methods for grouped survival data were conducted within the cohort to compute relative risks (RRs) of ratios of SIRs, taking into account age (5-year age groups) at HL diagnosis, calendar year (5-year calendar periods) of HL diagnosis and time since HL diagnosis (5-year calendar periods) (Preston *et al*, 1993). This approach incorporates SEER primary BC IRs (specific for clinical and pathologic features) to account for the natural rise in BC risk with increasing age (Yasui *et al*, 2003).

Age-specific IRs (<15, 15–24, 25–34, 35–44, 45–54, 55–64, 65–74, 75–84 years) for first primary female BC in the general population diagnosed during 1990–2005 were calculated using the SEER^*^Stat Incidence Rate module. BC age-specific IRs were calculated among HL survivors diagnosed with HL before 35 years of age and treated with RT by dividing the number of BC cases diagnosed during 1990–2005 at specified attained ages (<15, 15–24, 25–34, 35–44, 45–54, 55–64, 65–74, 75–84 years) by the HL population at-risk. There were no cases of BC diagnosed before 15 years of age or at the age of 65 years and older among HL survivors. All IRs were expressed per 100 person-years and plotted on a log-linear scale as previously described (Devesa *et al*, 1995). IRs based on fewer than 5 cases were omitted from the figures.

## Results

Overall, 2645 young female 5-year survivors of HL diagnosed between 1973–2000 and treated with RT as part of initial therapy had a six-fold risk of developing invasive BC compared with that expected in the general population (SIR=6. 13, 95% CI=5.23–7.13; Obs.=166) (Table 1). Standardised incidence ratios decreased with increasing attained age; however, compared with those <35 years at BC diagnosis, statistically significant differences among older attained age groups were not observed when the RRs were adjusted for age at HL diagnosis. Most BCs were diagnosed after 1989, and risks were similar across calendar year periods (*P*_RR_>0.5). Ductal adenocarcinoma and upper outer quadrant tumours were diagnosed most frequently. Relative risks did not differ significantly according to histology or laterality.

In comparison with that expected for primary BC in the general population, there were no significant differences in risks for developing localised, regional or distant stage BC following RT for HL. Multivariate analyses comparing SIRs (while adjusting for age at HL diagnosis and time since HL diagnosis) did not demonstrate statistically significant differences in risk of BC for regional stage (RR=0.87) or distant stage (RR=0.88) disease compared with localised stage BC. We also considered the possibility that screening for subsequent radiation-related BC could increase the likelihood of detecting localised BC in the more recent calendar years, and therefore assessed differences in RT-related BC risk by stage over time (1990–1993, 1994–1997, 1998–2001, 2002–2005). There was no significant trend observed for localised (*P*_Trend_=0.7), regional (*P*_Trend_=0.6), distant (*P*_Trend_=1.0) or unknown (*P*_Trend_=0.9) stages according to calendar year groups. Among LN-negative BC diagnosed during 1990–2005, we observed somewhat lower risks for larger (>2 cm) than smaller (⩽2 cm) tumours; however, this difference was not statistically significant in multivariate RR analyses (*P*_RR_=0.12).

Irradiated HL survivors had a greater than nine-fold risk of developing ER-negative/PR-negative BC during 1990–2005 as compared with a nearly five-fold risk of ER-positive/PR-positive tumours. In age- and time-adjusted multivariate analyses, the RR of ER-negative/PR-negative disease was 66% higher than ER-positive/PR-positive BC (*P*_RR_=0.008). The risk of developing high-grade BC was modestly elevated, but did not differ significantly from risk of low-grade tumours (RR=1.26, *P*_RR_=0.19).

To assess for a possible radiation effect, we further evaluated hormone receptor status and grade according to time since HL diagnosis. The SIR of ER-negative/PR-negative BC and high-grade BC showed greater increases with time since HL diagnosis than did ER-positive/PR-positive or low-grade BC, respectively (Table 2). Among 15-year HL survivors, risk of receptor-negative BC was two-fold higher than receptor-positive disease (RR=1.99, *P*_RR_=0.002). Fifteen-year survivors also had a 52% significantly greater risk of high-grade compared with low-grade tumours (RR=1.52, *P*_RR_=0.03).

**Figure 1:**
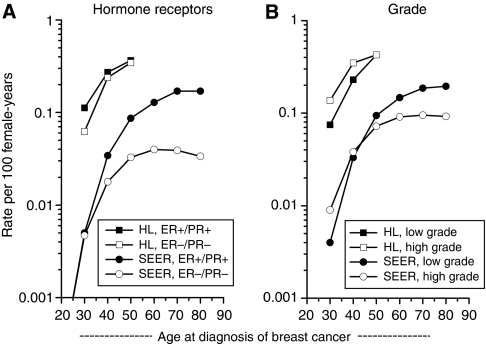
Age-specific incidence rates of female breast cancer (1990–2005) among 5-year survivors of Hodgkin's lymphoma (HL) diagnosed before 35 years of age and treated with radiotherapy and among the general population in nine registry areas of the Surveillance, Epidemiology and End Results (SEER) Programme, (**A**) according to oestrogen-receptor (ER) and progesterone-receptor (PR) status and (**B**) grade.

Age-specific BC IRs among HL survivors and primary BC IRs in the general SEER population are depicted in Figure 1. In contrast to divergent IRs with advancing age observed for ER-negative/PR-negative and ER-positive/PR-positive BC rates in the SEER population, IRs for receptor-negative BC among HL survivors approached those of receptor-positive disease as age increased (Figure 1A). In the general population, high-grade features predominated until approximately age 40 years, whereas low-grade BC was more common at older ages (Figure 1B). Among HL survivors, IRs of high-grade tumours exceeded that of low-grade tumours until approximately age 50 years.

## Discussion

This is the first study to demonstrate that young women treated with RT for HL have an increased risk of developing ER-positive/PR-positive and ER-negative/PR-negative BC compared with expected values in an age-matched series of BC in the general population. Overall, the RR of ER-negative/PR-negative BC was 66% higher than ER-positive/PR-positive BC among 5-year HL survivors, and nearly two-fold higher among 15-year survivors. In addition, 15-year survivors had a significant 50% greater risk of developing high-grade than low-grade BC compared with primary BC. Although longer follow-up is needed, temporal patterns suggest that radiation may contribute disproportionately to the development of BC with more adverse prognostic features than would be expected in the general population.

In contrast to our findings, after accounting for patient age, hospital-based case–control studies of BC among HL survivors have not found a significant variation in hormone-receptor status when compared with primary BC controls (Gaffney *et al*, 2001; Janov *et al*, 2001; Castiglioni *et al*, 2007). However, none of these studies considered time since HL diagnosis, and all were limited by small numbers of BC cases. A population-based study in North Carolina found a higher nonsignificant risk for ER-negative/PR-negative than ER-positive/PR-positive BC among women with a history of previous medical radiation to the chest compared with unexposed women, particularly at pre- and peri-menopausal ages (Huang *et al*, 2000). However, the study was based on fewer than 55 exposed cases, and risk according to time since exposure was not assessed.

Among HL survivors, higher SIRs for ER-negative/PR-negative BC than ER-positive/PR-positive BC may reflect lower baseline IRs for ER-negative/PR-negative BC in the general population. However, in our study, the incidence patterns by BC subtype among individuals with HL were distinct from those of primary BC. Whereas incidence of hormone receptor-positive BC in the general population exceeds that of ER-negative/PR-negative BC with advancing age, IR patterns by subtype among irradiated HL survivors revealed no such divergence. It is also important to consider that young women treated for HL may experience premature ovarian failure related to HL therapy (Trichopoulos *et al*, 1972; Fisher and Cheung, 1984); therefore, hormonal BC risk factors may differ from those in the general population. Additional follow-up of our study population will help clarify whether the risk patterns persist over time as HL survivors age and begin (or continue) to accumulate follow-up in the post-menopausal years.

Similar to others studies (Yahalom *et al*, 1992; Gaffney *et al*, 2001; Janov *et al*, 2001; Castiglioni *et al*, 2007; Sanna *et al*, 2007), we did not observe significant differences in overall risk of high-grade and low-grade tumours. However, with increasing time since HL diagnosis, risk of high-grade BC exceeded that of low-grade BC, suggesting a radiation effect. Although this observation has not been reported in other studies of HL survivors compared with hospital controls, none have considered time since HL diagnosis (Yahalom *et al*, 1992; Gaffney *et al*, 2001; Janov *et al*, 2001; Castiglioni *et al*, 2007; Sanna *et al*, 2007).

Other studies of HL survivors (Wolden *et al*, 2000; Castiglioni *et al*, 2007) have found that radiation-related BCs have a similar stage distribution to that of primary BC, which is consistent with our findings. A Stanford-based investigation suggested a higher proportion of early-stage BC among women treated for HL after 1990, perhaps due to increasing use of mammography (Wolden *et al*, 2000). However, many long-term HL survivors remain unaware of their increased risk of BC (Diller *et al*, 2002), and a recent survey of childhood cancer survivors treated with RT reported much lower BC screening rates than recommended (Oeffinger *et al*, 2009).

The strengths of our study include the large number of second BCs occurring among nearly 2700 irradiated HL survivors diagnosed over a 30-year period. A unique feature is the population-based study design, which compared BC following HL to that expected in the general population using age-matched, subtype-specific BC IRs. Despite decentralised hormone receptor testing in various laboratories, SEER data have been shown to be reasonably reliable for ER-positive/PR-positive and ER-negative/PR-negative BC subtypes (Ma *et al*, 2009). Limitations include the lack of detailed information on RT dose, additional HL treatments and other established BC risk factors.

Our population-based study suggests that long-term survivors of HL treated with RT before 35 years of age have a significantly higher risk of developing ER-negative/PR-negative than ER-positive/PR-positive BC, particularly among 15-year survivors. Fifteen-year HL survivors also had a significantly higher risk of developing high-grade than low-grade tumours. Although confirmatory studies are needed, the temporal patterns we observed suggest that radiation may contribute disproportionately to the development of BC with adverse prognostic features.

## Figures and Tables

**Table 1:** Risk of invasive breast cancer among 2645 female 5-year survivors (32 731 person-years) of Hodgkin's lymphoma diagnosed before age 35 years, initially treated with radiotherapy, and reported to the SEER-9 Programme, 1973–2005

**BC characteristics**	**Obs.**	**SIR**	**(95% CI)**	**RR^a^**	**(95% CI)**	***P*_RR_**
Total	166	6.13	(5.23–7.13)	NA	NA	NA
*Attained age (years)*						
<35	33	15.54	(10.70–21.83)	1.00	NA	
35–44	82	6.68	(5.31–8.29)	0.70	(0.47–1.08)	0.10
⩾45	51	4.02	(2.99–5.28)	0.66	(0.40–1.09)	0.10
*Attained calendar year*						
1978–1989	11	4.69	(2.34–8.40)	1.00	NA	
1990–2005	155	6.26	(5.32–7.33)	1.05	(0.59–2.06)	>0.5
*Histology* b						
Ductal adenocarcinoma	131	6.45	(5.39–7.65)	1.00	NA	
Lobular carcinoma	9	5.47	(2.50–10.39)	1.00	(0.47–1.86)	>0.5
Medullary carcinoma	7	15.49	(6.23–31.92)	2.17	(0.91–4.32)	0.08
Other or not specified	19	4.06	(2.44–6.34)	0.65	(0.39–1.03)	0.07
*Site* c						
Upper outer quadrant	82	8.30	(6.60–10.31)	1.00	NA	
Lower outer quadrant	10	5.78	(2.77–10.63)	0.70	(0.34–1.28)	0.26
Upper inner quadrant	5	1.96	(0.64–4.56)	0.24	(0.08–0.53)	<0.001
Lower inner quadrant	8	6.32	(2.73–12.46)	0.79	(0.35–1.54)	>0.5
Central/nipple	8	5.43	(2.34–10.70)	0.68	(0.30–1.31)	0.27
Overlapping	30	5.50	(3.71–7.86)	0.66	(0.43–1.00)	0.048
Not specified	23	4.85	(3.07–7.27)	0.58	(0.36–0.91)	0.02
*Laterality*						
Right	84	6.28	(5.01–7.77)	1.00	NA	
Left	82	6.04	(4.81–7.50)	0.96	(0.71–1.31)	>0.5
*Stage*						
Localised	97	6.31	(5.12–7.70)	1.00	NA	
Regional	58	5.89	(4.47–7.61)	0.87	(0.62–1.20)	0.39
Distant	8	5.84	(2.52–11.50)	0.88	(0.39–1.70)	>0.5
Not specified	3	6.07	(1.25–17.74)	0.84	(0.21–2.24)	>0.5
*Limited to breast cancer cases diagnosed 1990–2005* d						
*Tumour size and LN*^e^						
⩽2 cm and LN negative	63	6.40	(4.92–8.19)	1.00	NA	
>2 cm and LN negative	16	4.62	(2.64–7.51)	0.66	(0.37–1.11)	0.12
⩽2 cm and LN positive	24	6.62	(4.24–9.85)	0.96	(0.59–1.52)	>0.50
>2 cm and LN positive	25	5.50	(3.56–8.12)	0.76	(0.47–1.20)	0.24
*Grade*^f^						
Low	57	5.02	(3.80–6.51)	1.00	NA	
High	75	7.33	(5.77–9.19)	1.26	(0.89–1.80)	0.19
Not specified	23	7.25	(4.60–10.89)	1.34	(0.81–2.16)	0.25
*ER/PR*						
Positive/positive	63	4.96	(3.81–6.35)	1.00	NA	
Positive/negative	12	6.23	(3.22–10.88)	1.24	(0.64–2.22)	>0.50
Negative/positive	1	1.31	(0.03–7.30)	0.24	(0.01–1.10)	0.07
Negative/negative	51	9.31	(6.93–12.25)	1.66	(1.14–2.41)	0.008
Other and unspecified^g^	28	7.21	(4.79–10.43)	1.38	(0.87–2.13)	0.17

Abbreviations: BC=breast cancer; CI=confidence interval (exact); ER=oestrogen receptor; HL=Hodgkin's lymphoma; LN=lymph nodes; NA=not applicable; Obs=observed number of breast cancers; PR=progesterone receptor; RR=relative risk; SEER=Surveillance, Epidemiology and End Results; SIR=standardised incidence ratio.

a Attained calendar year and attained age are adjusted for age at HL diagnosis. All other analyses are adjusted for age at HL and time since HL diagnosis.

b Ductal adenocarcinoma includes International Classification of Diseases for Oncology, third edition (ICD-O-3) morphology codes M8500 and M8501, lobular carcinoma includes M8520 and M8521, and medullary carcinoma includes M8510–8512.

c ICD-O-3 topography codes include upper outer quadrant (C504, C506), lower outer quadrant (C505), upper inner quadrant (C502), lower inner quadrant (C503), central/nipple (C500–501), overlapping (C508), and not specified (C509).

d Includes 2549 female 5-year survivors of HL initially treated with radiotherapy who developed subsequent breast cancer (Obs.=155).

e Limited to localised and regional stage disease. Excludes 27 women with distant or unknown stage (*n*=10) and localised or regional stage disease without known tumour size and/or lymph node involvement (*n*=17).

f Grade was considered in three categories: low (grades I and II), high (grades III and IV) and unspecified.

g Includes ‘testing not done’, ‘borderline’ and ‘unknown’ ER and/or PR status.

**Table 2:** Risk of invasive breast cancer occurring in 1990 or later among 2549 female 5-year survivors of Hodgkin's lymphoma diagnosed before 35 years of age and treated with initial radiotherapy diagnosed in the SEER-9 Programme (1973–2005)

	**Time since Hodgkin lymphoma**																	
	**5–9 years**						**10–14 years**						**⩾15 years**					
**Breast cancer characteristics**	**Obs.**	**SIR**	**(95% CI)**	**RR** a	**(95% CI)**	**P_RR_**	**Obs.**	**SIR**	**(95% CI)**	**RR** a	**(95% CI)**	**P_RR_**	**Obs.**	**SIR**	**(95% CI)**	**RR** a	**(95% CI)**	**P_RR_**
*Grade* b																		
Low	3	3.06	(0.63–8.94)	1.00	NA	0.28	10	4.75	(2.28–8.73)	1.00	NA	>0.5	44	5.32	(3.87–7.15)	1.00	NA	0.03
High	2	1.47	(0.18–5.32)	0.37	(0.05–2.30)		13	5.63	(3.00–9.63)	1.07	(0.46–2.53)		60	9.14	(6.98–11.77)	1.52	(1.03–2.26)	
*ER/PR*																		
Positive/positive	3	2.46	(0.51–7.19)	1.00	NA	0.48	12	4.77	(2.47–8.34)	1.00	NA	0.44	48	5.35	(3.95–7.09)	1.00	NA	0.002
Negative/negative	1	1.32	(0.03–7.37)	0.46	(0.02–3.69)		9	7.21	(3.30–13.69)	1.42	(0.57–3.39)		41	11.81	(8.48–16.02)	1.99	(1.30–3.02)	

Abbreviations: BC=breast cancer; CI=confidence interval (exact); ER=oestrogen receptor; NA=not applicable; Obs.=observed number of breast cancers; PR=progesterone receptor; RR=relative risk; SEER=Surveillance, Epidemiology and End Results; SIR=standardised incidence ratio.

a Adjusted for age at Hodgkin's lymphoma diagnosis.

b Low grade includes grades I and II, and high grade includes grades III and IV.
